# Gastrointestinal dysfunction in patients with Mowat–Wilson syndrome is associated with feeding difficulties and altered plasma neurotransmitters

**DOI:** 10.3389/fped.2026.1751767

**Published:** 2026-04-10

**Authors:** Lingya Liu, Lihua Wu, Ruijie Zhou, Zhen Zhang, Ping Xiao, Bo Li, Qi Li, Qian Jiang, Jianxin Wu

**Affiliations:** 1 Department of Pediatrics, Beijing Tongren Hospital, Capital Medical University, Beijing, China; 2 Department of Medical Genetics, Capital Center for Children's Health, Capital Medical University, Capital Institute of Pediatrics, Beijing, China; 3 Department of General Surgery, Capital Center for Children's Health, Capital Medical University, Capital Institute of Pediatrics, Beijing, China; 4 Department of Pathology, Capital Center for Children's Health, Capital Medical University, Capital Institute of Pediatrics, Beijing, China; 5 Translational Medicine Program, The Hospital for Sick Children, Toronto, ON, Canada

**Keywords:** feeding difficulties, intestinal dysfunction, Mowat–Wilson syndrome, neurotransmitter, serotonin

## Abstract

**Background:**

Intestinal dysfunction is prevalent in children with Mowat–Wilson syndrome (MWS), yet its underlying mechanisms remain unclear. This study aimed to characterize intestinal symptoms, feeding patterns, and fasting plasma neurotransmitter profiles in patients with MWS, and to explore their potential relationships.

**Methods:**

Three complementary assessments were conducted, including a questionnaire assessing defecation difficulties and stool characteristics; a structured questionnaire assessing feeding difficulties, caregiver-reported dietary composition (including the proportion of meat), and complementary feeding practices; and targeted fasting plasma neurotransmitter profiling using UPLC–TQ–MS.

**Results:**

Among 35 patients with MWS, 86% had intestinal symptoms, including constipation (69%) and Hirschsprung disease (17%). In the feeding/diet analysis, 44.4% (4/9) of patients with MWS reported feeding difficulties. Compared with age-matched healthy controls (*n* = 10), patients with MWS (*n* = 9) had a significantly lower proportion of meat and delayed introduction of meat-based complementary foods (10.5 months vs. 7.9 months). In the plasma analysis, patients with MWS (*n* = 6) exhibited significantly reduced plasma levels of serotonin and taurine, alongside elevated levels of GABA and dopamine, compared with age-matched healthy controls (*n* = 10).

**Conclusion:**

These findings confirm the high prevalence of constipation-predominant intestinal dysfunction in patients with MWS. Collectively, our findings support further investigation of associations among feeding difficulties, a lower proportion of meat in dietary composition, and an altered fasting plasma neurotransmitter profile (including lower serotonin in a small subset), which may be relevant to gut dysmotility in patients with MWS. This diet–neurotransmitter axis offers a working model for understanding intestinal dysfunction in patients with MWS, yet direct quantification of dietary and circulating tryptophan in future studies is needed to validate this pathway.

## 1 Introduction

Mowat–Wilson syndrome (MWS) is a rare genetic disorder caused by heterozygous variants or deletions in the *ZEB2* gene. Patients with MWS show marked phenotypic variability, and gastrointestinal involvement is common, ranging from Hirschsprung disease (HSCR) to persistent constipation and other motility-related symptoms (1–3). Reported cohorts indicate substantial rates of HSCR and chronic constipation among patients with MWS (4). Indeed, one analysis suggested that the combined frequency of HSCR and chronic constipation among patients with MWS exceeds 73% (4, 5). Additionally, we observed that compared with patients with isolated HSCR, patients with MWS who had HSCR experienced a significantly higher rate of constipation recurrence after surgical correction of HSCR (6). However, the mechanisms underlying the high prevalence and persistence of intestinal dysfunction in patients with MWS remain unclear. Clinically, caregivers frequently report feeding difficulties in patients with MWS, including picky eating and reluctance to consume meat; yet feeding-related phenotypes have rarely been characterized systematically in this population.

These observations led us to consider a diet-related pathway involving serotonin (5-hydroxytryptamine, 5-HT), a key regulator of gut motility. Dietary tryptophan is the essential precursor for 5-HT synthesis, and intestinal enterochromaffin cells are a major source of peripheral 5-HT (7). Tryptophan cannot be synthesized by humans and must be obtained from foods such as poultry, red meat, fish, eggs, dairy products, and oats. While plant-based proteins also provide tryptophan, recent studies have shown that tryptophan derived from animal protein is significantly more bioavailable (7, 8). In the gut, tryptophan is the rate-limiting precursor for 5-HT production: enterochromaffin (EC) cells and certain enteric neurons hydroxylate tryptophan to 5-hydroxytryptophan (5-HTP), which is subsequently decarboxylated to generate 5-HT; released serotonin modulates intestinal motility, secretion, and vasodilation (9–12). Altered serotonergic signaling contributes to disordered gastrointestinal motility in multiple functional bowel conditions, but the status of circulating 5-HT in patients with MWS and its relationship to constipation-predominant dysfunction have not been investigated (13–16).

In the current study, we systematically evaluated intestinal function, feeding behavior, and neurotransmitter levels in patients with MWS. We hypothesized that feeding difficulties and a lower proportion of meat in caregiver-reported dietary composition, used as an indicator of meat-containing dietary patterns, may be associated with altered neurotransmitter levels and constipation-predominant intestinal dysfunction in patients with MWS. We next used a standardized feeding difficulty scale and a dietary survey to characterize feeding problems, such as limited meat intake and delayed introduction of meat-based complementary foods, in patients with MWS and compared the findings with data from age-matched healthy controls. Additionally, we measured the plasma concentrations of key neurotransmitters [including 5-HT, *γ*-aminobutyric acid (GABA), and dopamine] in patients with MWS and age-matched healthy controls. Through this multifaceted approach, we aimed to characterize intestinal symptoms, feeding patterns, and plasma neurotransmitter profiles in patients with MWS, and to explore potential associations among these features, thereby generating hypotheses for future mechanistic and interventional studies.

## 2 Methods

### 2.1 Participants and clinical data collection

Patients with genetically confirmed Mowat–Wilson syndrome were recruited from the Capital Institute of Pediatrics between June 2023 and December 2023. All participants had previously been diagnosed at the institute. The inclusion criteria were as follows: (1) clinical features consistent with MWS (distinctive facial gestalt and at least one congenital anomaly involving the nervous, cardiovascular, intestinal or genitourinary system) and (2) a pathogenic variant in *ZEB2* identified by next-generation sequencing and confirmed by Sanger sequencing.

The study protocol was approved by the Ethics Committee of the Capital Institute of Pediatrics (approval No. SHERLL2022023), and written informed consent was obtained from the parents or legal guardians of all participants.

Caregivers completed a structured questionnaire under clinician supervision, using the same protocol as in a previous study (6). The instrument first captured baseline clinical characteristics and multisystem comorbidities, including demographic data (age, sex, date of birth), the presence of congenital anomalies such as Hirschsprung disease, congenital heart defects, urogenital malformations, and any postoperative complications and their management. Subsequently, the questionnaire focused on intestinal function; the documentation of bowel movement frequency and stool consistency; defecation awareness; the ability to voluntarily delay defecation; episodes of fecal soiling or incontinence; constipation severity; patterns of abdominal distension; and diarrheal features (e.g., mild, explosive, or bloody stools) and used predefined categorical scales to enhance data uniformity and enable reproducible analysis.

All patients with MWS and HSCR included in this study had undergone definitive pull-through surgery with resection of the aganglionic bowel segment prior to enrollment; accordingly, the subgroup of patients with MWS and HSCR was assessed in the postoperative setting after removal of the aganglionic segment. Surgical characteristics and postoperative bowel function were recorded for patients with MWS and HSCR who underwent pull-through surgery. Constipation severity and motility-related symptoms were graded using predefined categories based on bowel movement frequency, stool form, and need for dietary or pharmacological intervention as captured in the questionnaire. Additional feeding and diet assessments were performed in a subset of enrolled participants.

### 2.2 Survey on feeding difficulty scores and the feeding status of the participants

Feeding difficulties were assessed using an adapted Chinese version of the Montreal Children's Hospital Feeding Scale (MCHFS) in patients with MWS in the feeding/diet substudy and in age-matched healthy controls with no reported history of chronic constipation, Hirschsprung disease, chronic/recurrent enteritis, gastrointestinal surgery, or developmental disorders (17). The modified MCHFS consists of 14 items, each rated on a seven-point Likert scale (1 = no difficulty to 7 = severe difficulty). Raw scores were converted to T-scores using a logit transformation as described by Ramsay. Participants were classified as having mild (T = 61–65), moderate (T = 66–70), or severe (T > 70) feeding difficulties.

Dietary diversity and composition were assessed using a caregiver-completed dietary questionnaire developed for this study (Supplementary Table S1). The caregivers reported whether each of the 12 food groups (including cereals/grains, legumes, meats, fruits, vegetables, and dairy products) had been consumed by the child during the previous week. To assess daily intake, caregivers were asked to recall the proportional consumption from four major food groups (meat, fruits and vegetables, dairy products, and grains) on the day before the survey. The questionnaire also recorded the age (in months) at which each of these four food categories was first introduced as complementary food during infancy, based on caregiver recall. Caregivers were assisted by trained staff when completing the feeding and dietary questionnaires to ensure clarity and consistency. This questionnaire captured proportional food composition (e.g., meat %) rather than absolute intake (grams/day). Total protein intake and dietary tryptophan intake (mg/day) were not calculated from dietary records. Therefore, meat proportion was treated as an indicator of dietary patterns containing animal protein, rather than as a quantitative estimate of protein or tryptophan intake. To enhance consistency during questionnaire completion, we employed a standardized instruction script, pre-specified food-group examples, and a mandatory check ensuring that the four food-group proportions summed to 100%.

Feeding and diet questionnaires were administered by trained research staff either in person or, when an in-person visit was not feasible, via telephone or video call, with a standardized instruction script used across all modes. Staff assistance was limited to: (1) explaining item meaning and response anchors; (2) providing pre-specified examples of food groups aligned with the questionnaire (e.g., poultry, livestock, and fish counted as “meat”); (3) checking for completeness and internal consistency, including ensuring that responses to the four food-group proportion items summed to 100%; and (4) resolving missing or inconsistent entries through neutral clarification, without prompting any particular answer. Caregivers reported the age of complementary food introduction (in months) based on recall. The full set of questionnaire items is provided in Supplementary Table S1.

### 2.3 Plasma sample collection and neurotransmitter analysis

Morning fasting venous blood samples were obtained from a subset of patients with MWS and age-matched healthy controls between June 2023 and December 2023. Approximately 2 mL of blood was collected into EDTA-coated vacuum tubes. To minimize potential confounding effects of medication, participants who had used medication within 1 month before fasting blood collection were excluded from the plasma substudy. The samples were maintained on ice and centrifuged within 2 h of collection at 3,000 × g for 10 min at 4 °C to isolate the plasma, which was subsequently aliquoted and stored at –80 °C until analysis.

Plasma neurotransmitter levels were quantified using a liquid mass platform (UPLC-TQ-MS) to detect neurotransmitter-related metabolites in biological samples (Metabo-Profile, Shanghai, P. R. China). Plasma samples were thawed on ice and precipitated with methanol containing internal standards. They were then vortexed, centrifuged, vacuum-dried, and derivatized using phenyl isothiocyanate (PITC). After nitrogen drying, the metabolites were reconstituted in an ammonium acetate–methanol solution and centrifuged, and the resulting supernatants were analyzed by UPLC–MS/MS (ACQUITY UPLC-Xevo TQS, Waters Corp., Milford, MA, USA). The sample run order was randomized, and all procedures were conducted in compliance with the ISO 9001 quality standards for sample and data handling. Raw mass spectrometry data were processed with Waters MassLynx (v4.1) for peak extraction, analyte identification, and quantification via calibration curves from serial dilutions. Subsequent multivariate analyses, namely principal component analysis (PCA) and orthogonal partial least squares discriminant analysis (OPLS-DA), were conducted using the iMAP platform (Metabo-Profile, Shanghai). To assess potential overfitting of the OPLS-DA model, a permutation test with 1000 random label permutations was performed, and R2Y/Q2 distributions and intercepts were examined.

### 2.4 Statistical analysis

All statistical analyses were performed using GraphPad Prism software (version 10.0; GraphPad Software, San Diego, CA, USA). Data distribution was assessed using the Shapiro–Wilk test. For normally distributed variables, data are presented as the mean ± standard deviation (SD) and compared between groups using an unpaired two-tailed Student's t test. For non-normally distributed variables, data are shown as medians with interquartile ranges (IQR) and compared using the two-tailed Mann–Whitney U test. Categorical variables were analyzed using the *χ*^2^ test or Fisher's exact test, as appropriate. A *P* value < 0.05 (two-sided) was considered statistically significant.

## 3 Results

### 3.1 Clinical characteristics of intestinal dysfunction in patients with MWS

**Figure 1:**
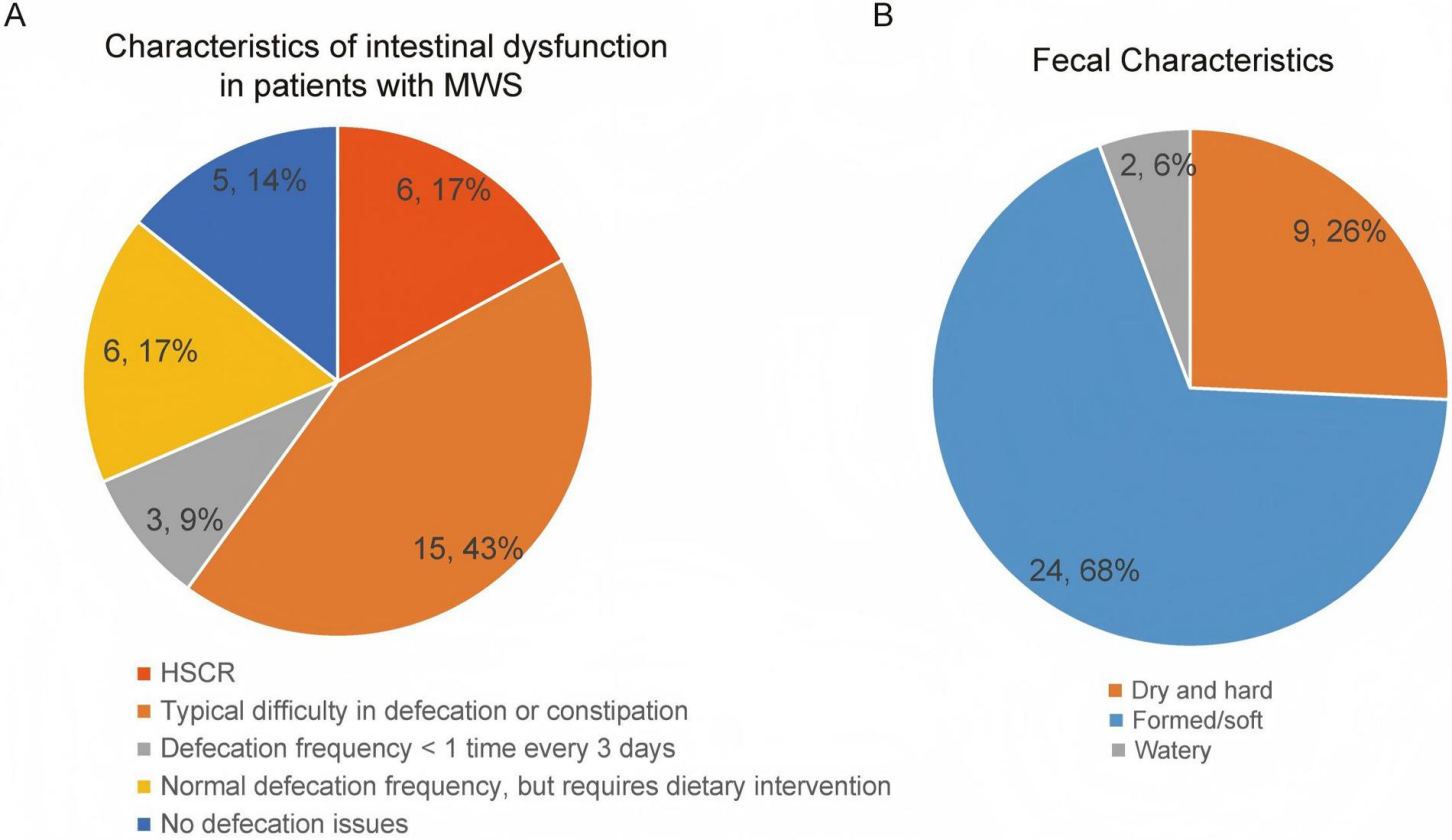
Characteristics of the intestinal dysfunction phenotype in patients with MWS. **(A)** Distribution of intestinal dysfunction categories: HSCR (17%, red), typical difficulty in defecation or constipation (43%, orange), defecation frequency <1 time per 3 days (9%, gray), normal frequency requiring dietary intervention (17%, yellow), and no defecation issues (14%, blue); **(B)** most patients with MWS experience constipation-predominant dysfunction: dry and hard (26%, orange), formed/soft (68%, blue) and watery (6%, gray) stool characteristics.

A total of 35 patients with MWS were included in the gastrointestinal questionnaire cohort. We first analyzed the clinical phenotypes of the recruited patients with MWS. In this cohort (median age 5 years, IQR 5–7; 54.3% female), all individuals presented with intellectual disability (100%), and nearly all exhibited delays in motor (97.1%) and language (91.4%) development. Notably, 85.7% (30/35) reported GI dysfunction. Additional features included a distinctive facial appearance (77.1%), seizures (51.4%), congenital heart disease (37.1%), genitourinary anomalies (25.7%), and microcephaly (20%) (Table 1). Intestinal dysfunction in these patients was further classified into HSCR (17% of patients), typical constipation (43%), infrequent defecation (occurring less than once every 3 days; 9%), and a normal defecation frequency achieved only with dietary intervention (17%), with the remaining 14% having no defecation issues (Figure 1A). Stool consistency patterns corroborated these findings; consistent with these intestinal manifestations, stool consistency was predominantly formed or soft (68% of patients), with dry or hard stools observed in 26% and watery stools in 6% (Figure 1B). Overall, these results highlight that constipation-predominant bowel dysfunction is the most common intestinal manifestation in our patients with MWS and is frequently accompanied by other developmental and congenital anomalies. Surgical characteristics and postoperative bowel function in patients with MWS and HSCR who underwent pull-through surgery are summarized in Supplementary Table S2.

**Table 1:** Clinical characteristics of patients with MWS in the current study.

Characteristic	*n* = 35	*n* (%) or median (IQR)
Age (years), median (IQR)	—	5 (5, 7)
Age group	—	—
3–5 years	19	54.3%
6–9 years	15	42.9%
≥10 years	1	2.9%
Gender		
Female	19	54.3%
Male	16	45.7%
Clinical phenotypes		
Intellectual disability	35	100.0%
Motor developmental delay	34	97.1%
Language delay	32	91.4%
Intestinal dysfunction	30	85.7%
Distinctive facial features	27	77.1%
Seizures	18	51.4%
Congenital heart disease	13	37.1%
Genitourinary anomalies	9	25.7%
Microcephaly	7	20.0%

### 3.2 Feeding difficulties, lower meat proportion, and delayed introduction of meat-based complementary foods

**Figure 2:**
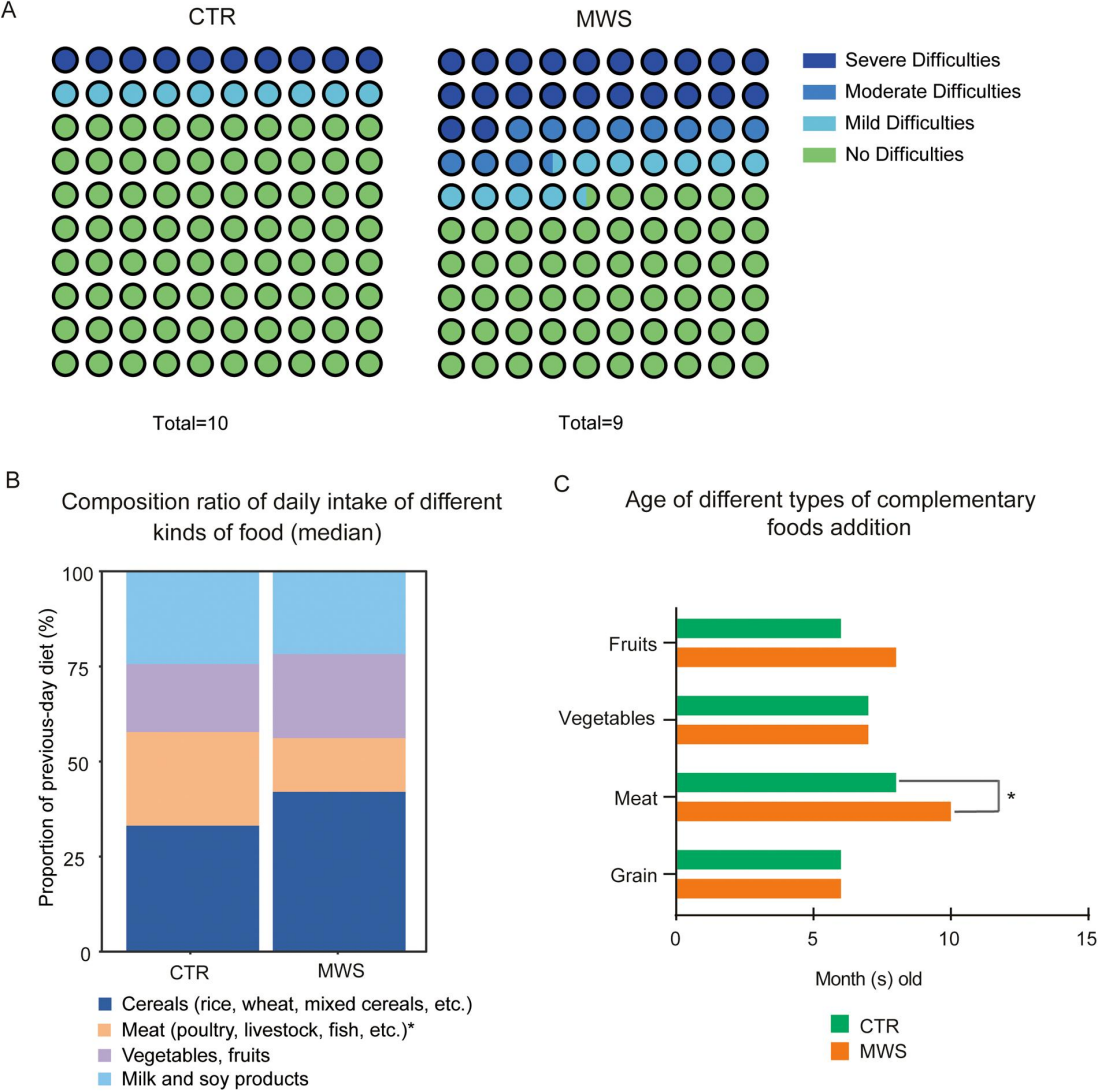
Feeding difficulty and dietary characteristics of patients with MWS (MWS) and age-matched healthy controls (CTR). **(A)** Percentage distribution of feeding difficulty severity in healthy controls (*n* = 10) and patients with MWS (*n* = 9). Colors denote no difficulty (green), mild difficulty (light blue), moderate difficulty (dark cyan) and severe difficulty (dark blue); **(B)** Composition of daily diet (median percentage) showing proportions of cereals (dark blue), meat (orange), vegetables/fruits (purple) and dairy/soy products (light blue). Patients with MWS consumed a smaller proportion of meat than age-matched healthy controls. Caregiver-reported proportions were required to sum to 100% (Supplementary Table S1); **(C)** Age at introduction of complementary foods (months). Meat was introduced later in patients with MWS (mean 10.5 months) than in age-matched healthy controls (mean 7.9 months), whereas fruits, vegetables and grains were introduced at similar ages. * indicates *P* < 0.05 (Mann–Whitney U test).

Among the gastrointestinal questionnaire cohort, 9 patients with MWS and 10 age-matched healthy controls completed the feeding/diet substudy. To evaluate potential selection bias, baseline characteristics and questionnaire-based gastrointestinal symptom measures were compared between substudy participants and nonparticipants (Supplementary Table S3). Feeding behavior was subsequently evaluated using an adapted Montreal Children's Hospital Feeding Scale. Demographic characteristics of the shared, age-matched healthy control cohort are summarized in Supplementary Table S4. Overall, we did not observe consistent differences across age, HSCR proportion, or most GI severity indicators; however, hard stool was more frequently reported among participants, raising the possibility that families with more prominent constipation-related stool form were more likely to complete additional assessments. Four of the nine patients with MWS (44.4%) reported feeding difficulties (one mild, one moderate, and two severe), whereas only two of the ten healthy controls (20%) reported difficulties (Figure 2A). Item-level analysis of the adapted MCHFS suggested that feeding difficulties in patients with MWS were mainly driven by higher caregiver-reported concerns and greater mealtime management challenges; detailed item scores are provided in Supplementary Table S5. Analysis of dietary intake diversity (12 food groups assessed over the previous week) revealed no differences between the two groups of individuals (Supplementary Figure S1). However, the proportion of meat in the previous day's diet was significantly lower in patients with MWS (median ∼20%) than in healthy controls (median ∼30%), while the intake of cereals, fruits/vegetables, and dairy/soy was comparable between groups (Figure 2B). Assessment of complementary feeding practices revealed that fruits, vegetables, and grains were introduced at similar ages (∼6–8 months) in both groups; however, meat-based complementary foods were introduced later in patients with MWS (mean 10.5 months, range 6–18 months) than in age-matched healthy controls (mean 7.9 months, range 6–15 months), a difference that reached statistical significance (Figure 2C, *P* = 0.03). Together, these findings indicate differences in feeding behavior and meat-related dietary patterns between groups. However, because the questionnaire captured proportional composition rather than absolute intake, these results should be interpreted as exploratory indicators rather than quantitative evidence of reduced animal protein or tryptophan intake.

### 3.3 Altered fasting plasma neurotransmitter profile in an exploratory subset of patients with MWS

**Figure 3:**
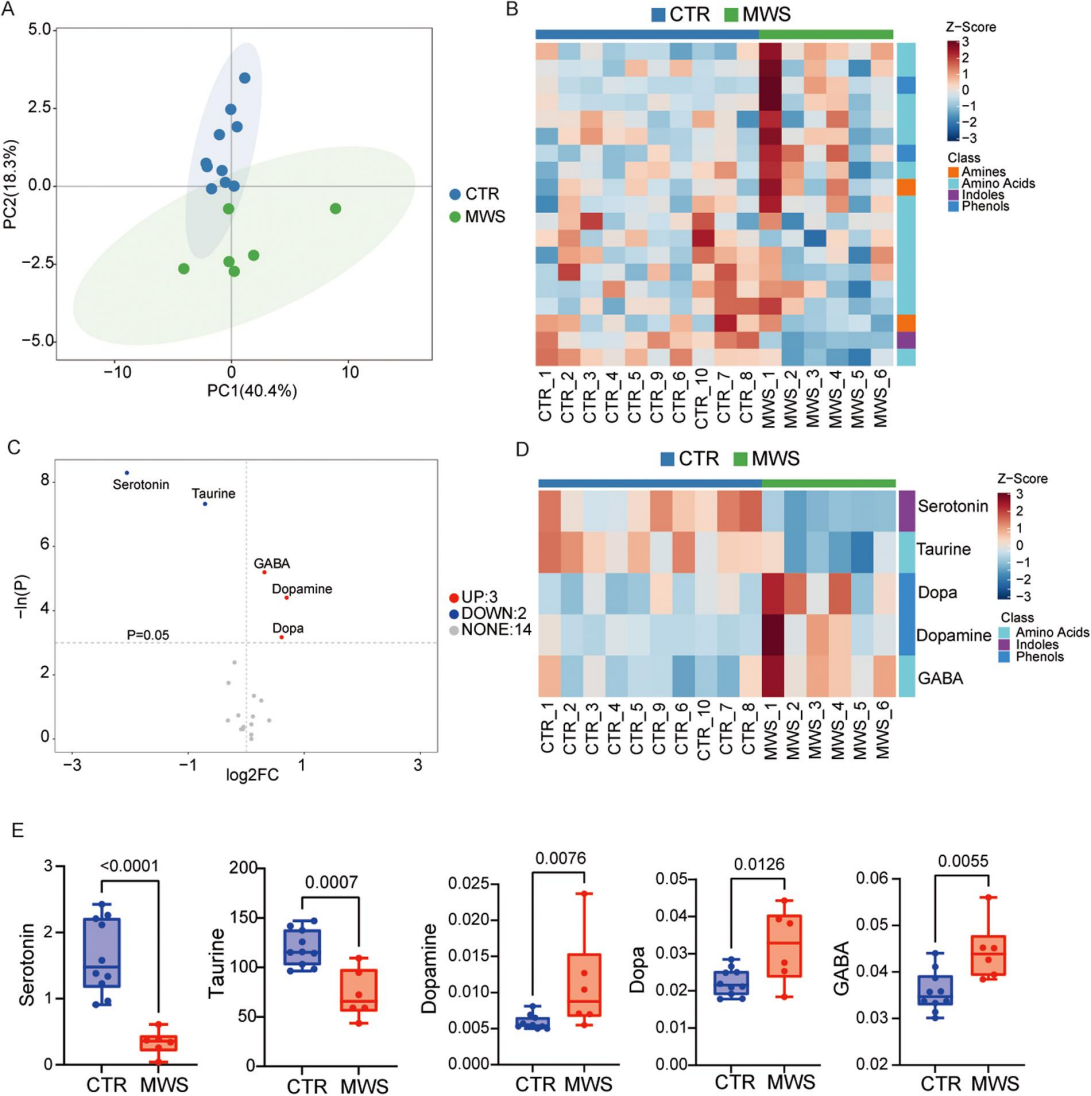
Plasma neurotransmitter profiling in patients with MWS (MWS) and age-matched healthy controls (CTR). **(A)** Principal component analysis (PCA) of 24 plasma neurotransmitters in the age-matched healthy controls (*n* = 10) and patients with MWS (*n* = 6); ellipses indicate 95% confidence intervals. **(B)** Heatmap of Z-scored concentrations of 24 neurotransmitters grouped by chemical class (amines, amino acids, indoles and phenols). Each column represents an individual sample. **(C)** Volcano plot showing log_2_ fold changes (MWS/CTR) against –ln (P); the levels of serotonin and taurine were significantly reduced (blue), whereas those of GABA, dopamine and DOPA were elevated (red). Dashed lines indicate *P* = 0.05 and log_2_ fold change = 0. **(D)** Heatmap of the five significantly altered neurotransmitters, revealing lower serotonin and taurine levels and higher GABA, dopamine, and DOPA levels in patients with MWS. **(E)** Min-to-max boxplots of metabolite levels. Statistical tests: unpaired t-test or Mann–Whitney U as appropriate.

Targeted neurotransmitter profiling of plasma from 6 patients with MWS and 10 age-matched healthy controls revealed distinct neurochemical signatures. The same age-matched healthy control cohort was used for the plasma neurotransmitter analysis; demographic characteristics of this cohort are provided in Supplementary Table S4. The six plasma samples from patients with MWS were obtained from the feeding/diet substudy subsample (6 of 9 participants). Principal component analysis (PCA) of 24 neurotransmitters suggested an overall separation between groups along the first two principal components (Figure 3A). To further visualize supervised group separation, OPLS-DA was performed (Supplementary Figure S2A), and potential overfitting was assessed using a 1000-permutation test (Supplementary Figure S2B). A heatmap of Z-scored neurotransmitter levels indicated that the levels of several amines, amino acids, indoles, and phenols were altered in patients with MWS (Figure 3B). Volcano-plot analysis revealed that serotonin and taurine were significantly reduced in patients with MWS, whereas *γ*-aminobutyric acid (GABA), dopamine and DOPA were significantly elevated, with serotonin showing the greatest decrease (log_2_ fold change < –2) (Figure 3C, Supplementary Figure S2C). A focused heatmap confirmed consistently lower serotonin and taurine levels and higher GABA and dopamine in patients with MWS than in age-matched healthy controls (Figures 3D, E). Collectively, these data indicate that patients with MWS exhibit a distinct plasma neurotransmitter profile characterized by reduced plasma serotonin levels (and altered GABA/catecholamines).

## 4 Discussion

MWS was first characterized in 1998 based on a series of six pediatric cases, five with HSCR and one with chronic constipation, thus indicating early recognition of intestinal involvement in this syndrome (3, 18). Subsequent studies revealed that approximately 43%–57% of patients with MWS present with HSCR, and those who undergo corrective surgery frequently experience recurrent postoperative constipation at rates significantly higher than those of patients with isolated HSCR (5, 6, 19). Moreover, individuals with MWS whose rectal mucosal biopsies lack evidence of aganglionosis (no confirmed HSCR) frequently experience persistent disease, underscoring the existence of functional motility abnormalities beyond structural pathology refractory to chronic constipation (5). In our cohort of 35 patients with MWS, 86% exhibited intestinal dysfunction, most commonly constipation-predominant bowel disturbances, confirming the high clinical prevalence observed in prior case series. This concordance between historical reports and our clinical data underscores the prevalence of intestinal dysfunction in patients with MWS but the absence of an established mechanistic explanation. Building on these clinical findings, this study offers a novel perspective by combining caregiver-reported feeding assessments with plasma neurotransmitter profiling. Our data highlight a high prevalence of constipation-predominant intestinal dysfunction in patients with MWS and an association between feeding difficulties/reduced proportion of meat and an altered fasting plasma neurotransmitter profile (including lower serotonin) in a small subset. Given the cross-sectional design and small subsamples, causality cannot be inferred and should be tested in larger prospective studies.

The observed associations among feeding difficulties, reduced proportion of meat (as an indicator of animal-protein–containing dietary patterns), lower plasma serotonin, and constipation in this cross-sectional study support a working model for hypothesis generation. It is important to note that total protein intake, dietary tryptophan, and plasma tryptophan were not directly quantified in this study. Plasma serotonin can also be influenced by enterochromaffin cell function, microbiota-related tryptophan metabolism, and genetic variation in serotonergic pathways; these were not assessed here and therefore residual confounding cannot be excluded. Thus, while meat is recognized as a key source of bioavailable tryptophan critical for serotonin (5-HT) production, our dietary findings should be interpreted as indicators of a broader pattern rather than direct evidence of tryptophan insufficiency. Animal proteins generally provide more digestible indispensable amino acids than most plant sources, with higher true ileal digestibility/bioavailability reported for tryptophan, positioning meat as a critical contributor to 5-HT production (8). Within the gut, enterochromaffin cells and enteric neurons convert tryptophan to 5-HT via tryptophan hydroxylase (TPH) mediated catalysis (12, 20). Mechanical and chemical cues in the lumen, including mechanosensation and microbially derived metabolites, activate ion channels on EC cells (e.g., Piezo2 and TRPA1) to trigger 5-HT release, thereby linking luminal signals to peristalsis. Experimental loss or inhibition of 5-HT signaling slows gastric emptying and colonic transit, such as in TPH1 knockout mouse models, leads to impaired colonic emptying, reduced pellet propulsion, and diminished migrating motor complex activity (21). Conversely, activation of 5-HT receptors in the enteric nervous system, specifically 5-HT_3_ and 5-HT_4_ receptors located in the myenteric and submucosal plexuses, restores rhythmic peristalsis and mitigates disease symptoms (22, 23). Moreover, numerous studies demonstrate that 5-HT signaling is essential not only for motility but also for maintaining gut mucosal integrity and the development of dopaminergic neurons in the gut (24, 25). Together, these data are consistent with a working model in which feeding difficulties and a lower proportion of meat (as an indicator of meat-containing dietary patterns) may be associated with lower tryptophan availability and reduced plasma 5-HT levels, which could relate to impaired gut motility and broader gastrointestinal symptoms.

Beyond the reduction in 5-HT, the plasma profile in patients with MWS was characterised by elevated *γ*-aminobutyric acid (GABA) and catecholamines together with decreased taurine, consistent with a broader neurotransmitter disequilibrium that could contribute to gut dysfunction. GABA is a key neurotransmitter within the enteric nervous system that regulates gastrointestinal motility and secretion. Mechanistic studies show that GABAergic signalling is widely distributed in the gut and interfaces with major enteric transmitters to shape motor and secretory programs (26). In the small intestine, GABA modulates cholinergic neurotransmission in myenteric circuits: presynaptic GABA receptor activation can facilitate acetylcholine (ACh) release, whereas GABA signalling generally suppresses ACh output, thereby tuning excitatory drive in ileal networks (27). In the colon, many studies demonstrate GABA participation in the peristaltic reflex (28). Beyond motor control, GABA influences mucosal and immune pathways and is increasingly recognized as a potential therapeutic target in inflammatory bowel disease (IBD) (29). In addition to alterations in 5-HT signaling, other neurotransmitter changes, including elevated catecholamines (such as dopa and dopamine) and decreased taurine, likely contribute to gastrointestinal regulation. Dopamine, acting through enteric D_2_ receptors, potently inhibits motility in both the small intestine and colon; indeed, studies demonstrate that D_2_ receptor engagement suppresses acetylcholine release and slows transit time (30). Taurine plays a critical role in maintaining epithelial barrier integrity and limiting mucosal inflammation: experimental supplementation in models of colitis enhances expression of tight-junction proteins and attenuates inflammatory responses, whereas taurine deficiency exacerbates barrier dysfunction and colonic inflammation (31). Plasma neurotransmitters were assessed from a single morning fasting blood draw, which enhances pre-analytical consistency by minimizing short-term dietary effects. However, repeated sampling will be necessary to evaluate within-individual temporal variability and to validate these metabolites as stable biomarkers. Collectively, patients with MWS exhibit reduced plasma 5-HT levels; together with elevated catecholamines and lower taurine, these alterations may be related to refractory gastrointestinal dysfunction, although causality cannot be inferred from the present study.

These findings emphasize the clinical utility of structured feeding assessments in patients with MWS, not only to enable early recognition of feeding difficulties but also to guide timely nutritional interventions that could potentially improve related constipation. Beyond the context of MWS, the observed pattern of associations provides a broader framework for exploring the link between feeding difficulties, dietary protein intake, and gastrointestinal dysfunction. It suggests that similar strategies may be applicable to functional constipation and other conditions characterized by impaired feeding and chronic bowel dysfunction, thereby extending the clinical relevance of our observations.

Although our study provides an initial view of the association between feeding difficulties, serotonin metabolism, and intestinal dysfunction in patients with MWS, it is limited by a modest overall sample size and by small subsamples for key analyses (feeding/diet substudy *n* = 9/35; plasma neurotransmitter profiling *n* = 6/35). These subsamples were underpowered; therefore, estimates, particularly multivariate pattern separation, should be interpreted as exploratory and hypothesis-generating. Additional limitations include the cross-sectional design, reliance on caregiver-reported measures, and the absence of direct quantification of total protein intake, dietary tryptophan, and plasma tryptophan. In addition, circulating biogenic amines may exhibit temporal variability, including time-of-day and meal-related effects (32). Although all plasma samples were obtained as morning fasting blood draws to enhance pre-analytical consistency, each participant contributed only a single time point; therefore, within-individual variability could not be assessed. Accordingly, the plasma neurotransmitter findings should be interpreted as exploratory cross-sectional associations and warrant confirmation in larger prospective cohorts with repeated, standardized sampling. Questionnaire data were collected either in person or, when an in-person visit was not feasible, via telephone or video call. Although a standardized script and neutral clarification procedures were used across all modes to enhance consistency, caregiver recall may introduce some imprecision—particularly regarding the age of complementary food introduction and estimates of proportional intake over the prior day. These gaps reflect the broader challenges of conducting intervention-oriented research in a heterogeneous population of individuals with MWS. While we did not observe systematic differences between participants and nonparticipants across most GI severity metrics (Supplementary Table S3), the higher frequency of hard stool among participants suggests that families with more prominent constipation-related stool form may have been more likely to complete additional assessments; therefore, selection bias cannot be fully excluded. Moreover, HSCR-related surgical factors and postoperative functional variability may influence gastrointestinal outcomes (and potentially related biochemical readouts), and should be addressed in future larger, stratified cohorts. Future studies should prioritize larger, prospective cohorts and incorporate objective measures of intestinal motility, quantitative nutritional assessment, and direct biochemical profiling of tryptophan and related metabolites (ideally in circulation and relevant biospecimens) to clarify the mechanistic links among feeding difficulties, dietary patterns, and serotonin-associated dysmotility. Prospective interventional trials (e.g., dietary optimization or tryptophan-focused strategies) may further inform therapeutic guidance for patients with feeding difficulties and gastrointestinal dysfunction. Comparative studies across disorders featuring both feeding difficulties and constipation could also help determine whether reduced serotonergic tone secondary to impaired feeding represents a broader, actionable pathway beyond patients with MWS.

## 5 Conclusion

Intestinal dysfunction is highly prevalent in patients with MWS. Our findings are consistent with a hypothesized pathway in which feeding difficulties and a lower meat proportion (as an indicator of meat-containing dietary patterns) may be associated with an altered fasting plasma neurotransmitter profile (including lower serotonin in a subset) and constipation-predominant intestinal dysfunction; causality cannot be inferred from this cross-sectional study. These correlative observations support the consideration of enhanced nutritional guidance and GI monitoring in clinical care. Future research should aim to test this model and determine whether addressing these factors can improve gastrointestinal outcomes.

## Data Availability

The original contributions presented in the study are included in the article/Supplementary Material, further inquiries can be directed to the corresponding author.
