# Supplementary material for: Gastrointestinal dysfunction in patients with Mowat–Wilson syndrome is associated with feeding difficulties and altered plasma neurotransmitters
Source: Front Pediatr. 2026 Apr 10;14:1751767. doi: 10.3389/fped.2026.1751767 (PMC13106388; doi:10.3389/fped.2026.1751767)
Supplement: Supplementary file 1 [file Datasheet1.docx]

Supplementary Material

Gastrointestinal Dysfunction in Patients With Mowat–Wilson Syndrome Is Associated With Feeding Difficulties and Altered Plasma Neurotransmitters

Lingya Liu^1^, Lihua Wu^2^, Ruijie Zhou^3^, Zhen Zhang^3^, Ping Xiao^4^, Bo Li^5^, Qi Li^3^, Qian Jiang^2^, Jianxin Wu^1*^

1. Department of Pediatrics, Beijing Tongren Hospital, Capital Medical University, Beijing, China
2. Department of Medical Genetics, Capital Center for Children's Health, Capital Medical University, Capital institute of Pediatrics, Beijing, China
3. Department of General Surgery, Capital Center for Children's Health, Capital Medical University, Capital institute of Pediatrics, Beijing, China
4. Department of Pathology, Capital Center for Children's Health, Capital Medical University, Capital institute of Pediatrics, Beijing, China
5. Translational Medicine Program, The Hospital for Sick Children, Toronto, ON, Canada

Correspondence:

Jianxin Wu

E-mail: [jianxinwu_2000@163.com](mailto:jianxinwu_2000@163.com)

# Supplementary Figures and Tables

**Questionnaire for child feeding**

DATE:_______ NAME OF CHILD:_________

Your relationship with the child:_______ Who is responsible for the feeding of the child:_______

1. Number of meals per day: _______

2. Composition ratio of the following foods the child consumed within the last day (Please ensure the total adds up to 100%.):

Meat (poultry, livestock, fish, etc.) _______% Cereals (rice, wheat, mixed cereals, etc.) _______%

Vegetables, fruits _______% Milk and soy products _______%

3. Whether children have food allergy (please fill in the specific type if Yes): ○ Yes______ ○ No

4. Whether the child has taken the following foods and the number of times in the last week:

| Frequency  Food type | No | Yes, everyday | Yes, 3-6 day per week | Yes, ≤ 2 day per week |
| --- | --- | --- | --- | --- |
| Staple (rice, noodles, cereals and potatoes, etc.) |  |  |  |  |
| Milk (including milk powder, milk, yogurt, cheese, etc.) |  |  |  |  |
| Aquatic products (such as fish, shrimp, etc.) |  |  |  |  |
| Livestock meat (pork, mutton, beef, etc.) |  |  |  |  |
| Poultry (duck, chicken, etc.) |  |  |  |  |
| Egg |  |  |  |  |
| Vegetables and fruits |  |  |  |  |
| Nuts (walnuts, cashews, almonds, melon seeds, peanuts, etc.) |  |  |  |  |
| Bean products (such as bean curd, soymilk, etc.) |  |  |  |  |
| Animal viscera (liver, kidney, heart, etc.) and animal blood (chicken blood, duck blood, pig blood, etc.) |  |  |  |  |
| Snacks (expanded food, biscuits, candy, chocolate, etc.) |  |  |  |  |
| Drinks (carbonated drinks, fruit and vegetable juice, energy drinks, etc.) |  |  |  |  |

5. Age at first introduction of the following complementary foods (months):

Cereals：_______month age Meat：_______month age

Vegetables：_______month age Fruits：_______month age

**Supplementary Table S1. Questionnaire used to investigate the feeding characteristics of** **patients with MWS and age‑matched healthy controls**

“Meat” was defined as poultry, livestock meat, and fish/aquatic products. For the four-group proportional intake item, caregivers were required to report percentages summing to 100%.

| **Patient ID** | **Sex** | **Included in diet substudy** | **Age at enrollment (years)** | **Age at surgery (months)** | **Procedure** | **Resection extent** | **Postoperative enterocolitis** | **Anastomotic complications*** | **Perianal excoriation** | **Intestinal perforation** | **Recurrent constipation** | **Stool frequency (post-op)** | **Stool form (post-op)** | **Bowel/constipation management (post-op)** |
| --- | --- | --- | --- | --- | --- | --- | --- | --- | --- | --- | --- | --- | --- | --- |
| 9 | Male | No | 8 | 6 | Definitive pull-through | Rectum + partial sigmoid | Yes | No | No | No | Yes | Normal | Formed/pasty | Dietary modification |
| 13 | Male | Yes | 6 | 9.5 | Definitive pull-through | Rectum + sigmoid + descending + transverse colon | No | No | Yes | No | No | >3/day | Formed/pasty | No constipation treatment required |
| 17 | Male | No | 5 | 4.7 | Definitive pull-through | Total colon + partial small intestine | No | Yes | Yes | Yes | Yes | >3/day | Watery | Rectal enema required |
| 25 | Female | No | 9 | 14.7 | Definitive pull-through | Rectum + partial sigmoid | No | No | No | No | No | Normal | Formed/pasty | No constipation treatment required |
| 26 | Female | No | 5 | 3 | Definitive pull-through | Rectum + partial sigmoid | No | No | No | No | Yes | Normal | Formed/pasty | Rectal enema required |
| 29 | Female | Yes | 5 | 3 | Definitive pull-through | Rectum + partial sigmoid | No | Yes | No | No | No | Normal | Formed/pasty | No constipation treatment required |

**Supplementary Table S2. Surgical characteristics and postoperative bowel function of six patients with MWS and HSCR who underwent pull-through surgery**

This table summarizes demographics, surgical timing and extent, and postoperative functional status for six patients with MWS and HSCR who underwent definitive pull-through surgery. “Included in diet substudy” indicates whether the patient completed the dietary/feeding assessment. Age at surgery (months) was calculated from recorded birth and surgery dates. Enterocolitis indicates a history of postoperative enterocolitis as documented in medical records and/or reported by caregivers. ^*^Anastomotic complications include reported anastomotic infection, retraction, or bleeding.

| **Characteristic** | **Participants (diet/plasma substudies) (n = 9)** | **Nonparticipants (n = 26)** | **P value** |
| --- | --- | --- | --- |
| Age (years), median [IQR] | 5.0 [5.0–6.0] | 5.5 [5.0–7.8] | 0.381 |
| HSCR | 2/9 (22.2%) | 4/26 (15.4%) | 0.635 |
| Stool frequency <1 per 3 days | 3/9 (33.3%) | 6/26 (23.1%) | 0.665 |
| Abnormal stool frequency | 5/9 (55.6%) | 11/26 (42.3%) | 0.700 |
| Hard stool | 5/9 (55.6%) | 4/26 (15.4%) | 0.030 |
| Constipation requiring management (dietary and/or medical intervention) | 2/9 (22.2%) | 15/26 (57.7%) | 0.121 |
| Fecal soiling (any) | 2/9 (22.2%) | 14/26 (53.8%) | 0.135 |
| Fecal incontinence (any) | 2/9 (22.2%) | 7/26 (26.9%) | 1.000 |
| Limited bowel awareness (partial/none) | 4/9 (44.4%) | 15/26 (57.7%) | 0.700 |

**Supplementary Table S3. Comparison of baseline characteristics and questionnaire-based gastrointestinal severity between participants and nonparticipants in the diet/plasma substudies**

Participants were the subset of patients with MWS who completed the additional feeding/diet assessment and/or contributed plasma for neurotransmitter profiling (n = 9); nonparticipants were the remaining patients who only completed the GI questionnaire (n = 26). Age is presented as median [interquartile range (IQR)] and compared using the two-sided Mann–Whitney U test. Categorical variables are presented as n/N (%) and compared using two-sided Fisher’s exact tests. Among participants (n = 9), fasting plasma for targeted neurotransmitter profiling was available for six individuals. Abnormal stool frequency was defined as <1 bowel movement per 3 days or >3 bowel movements per day (caregiver-reported).

| **Characteristic** | **Patients with MWS**  **(feeding/diet substudy) (n = 9)** | **Age‑matched healthy controls (n = 10)** | **P value** |
| --- | --- | --- | --- |
| Age (months), median [IQR] | 30.8 [25.2–36.1] | 30.9 [21.5–38.7] | 0.903 |
| Female | 6/9 (66.7%) | 5/10 (50.0%) | 0.650 |
| Plasma sample available (neurotransmitter substudy) | 6/9 (66.7%) | 10/10 (100%) | — |
| Gastrointestinal disease history | 8/9 (88.9%) | 0/10 (0.0%) | <0.001 |
| Abnormal stool frequency* | 5/9 (55.6%) | 0/10 (0.0%) | 0.011 |
| Stool frequency <1 per 3 days | 3/9 (33.3%) | 0/10 (0.0%) | — |
| Stool frequency >3 per day | 2/9 (22.2%) | 0/10 (0.0%) | — |

**Supplementary Table S4.** **Demographic characteristics and gastrointestinal history are shown for participants in the feeding/diet substudy**

Among the nine participants with MWS, fasting plasma samples were available for six individuals. Age (months) was calculated as years × 12 + months + days/30 based on caregiver-reported age at questionnaire completion. Categorical variables are presented as n/N (%). P values were calculated using the Mann–Whitney U test for continuous variables and Fisher’s exact test for categorical variables (two-sided). “Plasma sample available” indicates whether fasting plasma was collected for targeted neurotransmitter profiling, all six plasma samples from participants with MWS were from these nine participants. “Gastrointestinal disease history” was defined as any caregiver-reported history of gastrointestinal disorders (e.g., chronic constipation, Hirschsprung disease, enteritis/colitis, or other chronic gastrointestinal conditions). Controls were eligible only if they had no reported history of chronic constipation, Hirschsprung disease, chronic/recurrent enteritis, or gastrointestinal surgery. *Abnormal stool frequency was defined as either <1 bowel movement per 3 days or >3 bowel movements per day.

| **MCHFS item (scored)** | **Patients with MWS (n = 9)**  **median [IQR]** | **Age‑matched healthy controls (n = 10)**  **median [IQR]** | **P value** |
| --- | --- | --- | --- |
| Q1 Difficulty feeding the child | 4 [3–4] | 2 [1–3] | 0.026 |
| Q2 Concern about child's feeding/eating | 4 [2–6] | 2 [1–2.8] | 0.021 |
| Q3 Appetite (reverse-coded) | 3 [2–4] | 2 [2–3.5] | 0.604 |
| Q4 Refusal during meal (reverse-coded) | 2 [1–3] | 2 [1.2–3.5] | 0.899 |
| Q5 Meal duration | 3 [3–4] | 3 [2.2–3] | 0.329 |
| Q6 Mealtime disruptive behavior | 2 [2–5] | 3.5 [3–5.8] | 0.405 |
| Q7 Nausea/vomiting to specific foods | 2 [1–3] | 2 [1–2.8] | 0.733 |
| Q8 Holding food without swallowing | 3 [2–3] | 3 [2–3.8] | 0.933 |
| Q9 Needs coaxing/chasing to eat | 4 [2–5] | 2 [2–3] | 0.580 |
| Q10 Force-feeding | 3 [2–3] | 2.5 [1.2–3] | 0.497 |
| Q11 Chewing/sucking ability | 5 [4–6] | 2 [1–3] | 0.016 |
| Q12 Growth status | 3 [3–5] | 1.5 [1–3] | 0.038 |
| Q13 Impact on caregiver-child relationship | 1 [1–3] | 1 [1–1.8] | 0.539 |
| Q14 Impact on family relationship | 1 [1–2] | 1 [1–2] | 0.887 |

**Supplementary Table S5. Item-level results of the adapted Montreal Children’s Hospital Feeding Scale (MCHFS) in patients with MWS and age‑matched healthy controls**

Values are presented as median [interquartile range, IQR]. P values were calculated using the two-sided Mann–Whitney U test and are reported descriptively, given the exploratory nature of the analyses and the small sample size. Higher scores indicate greater feeding difficulty, based on the standard scoring direction (including reverse-coding of positively framed items). These results should be considered exploratory and hypothesis-generating due to the modest sample size.


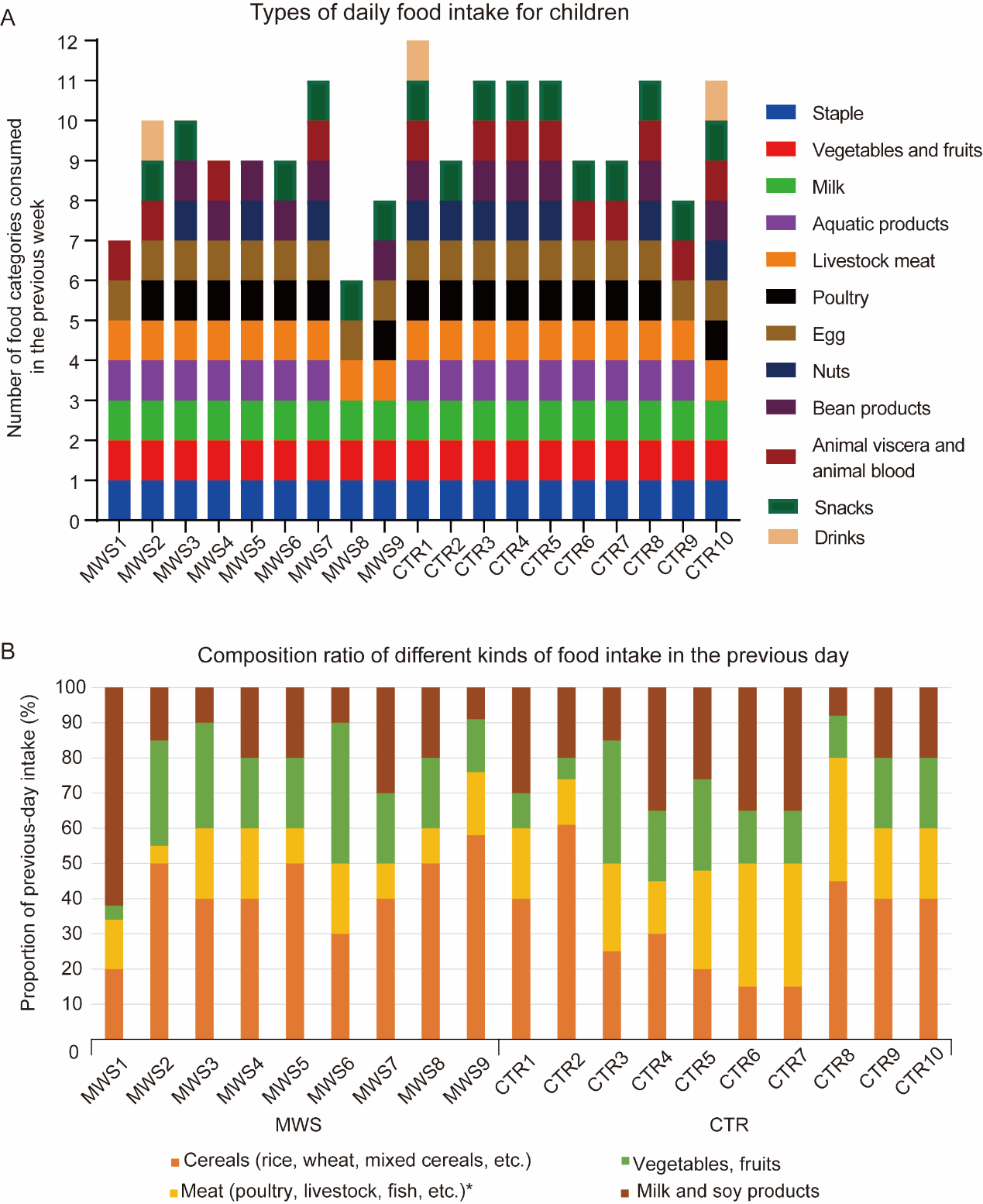


**Supplementary Fig S1. Daily dietary diversity and composition in patients with MWS (MWS) and age-matched healthy controls (CTR)**

A, Analysis of dietary diversity in patients with MWS (n = 9) and age-matched healthy controls (n = 10), including 12 categories of food. Each colored block indicates a food category consumed at least once during the previous week; total bar height reflects dietary diversity (number of categories). B, Composition of previous-day intake (%) showing caregiver-reported proportions of cereals, meat, vegetables/fruits, and dairy/soy products (required to sum to 100%). * P < 0.05.


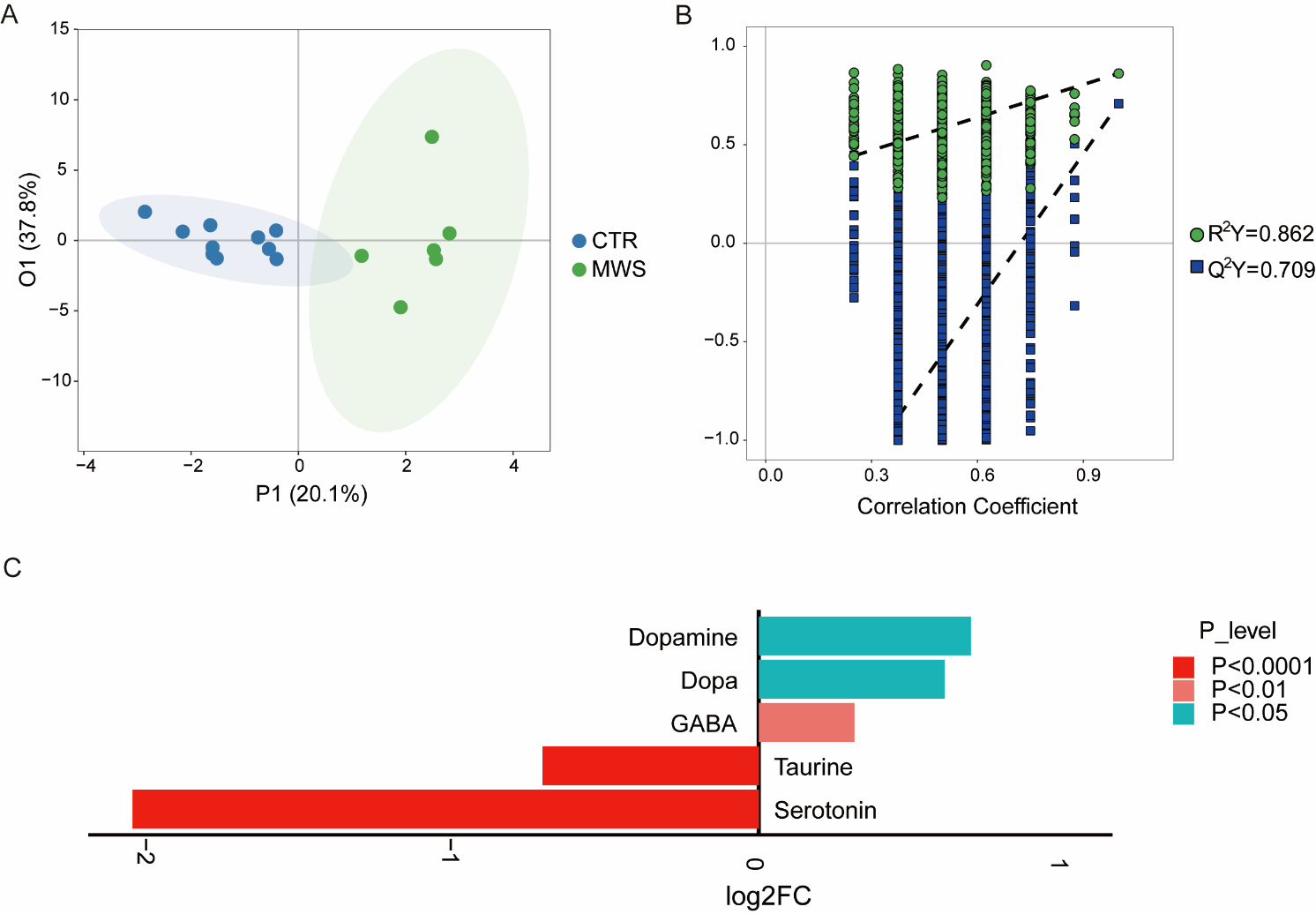


**Supplementary Fig S2. Multivariate assessment and differential plasma neurotransmitters in patients with MWS (MWS) and age-matched healthy controls (CTR)**

A, OPLS-DA score plot based on targeted plasma neurotransmitter profiling (UPLC–TQ–MS) comparing patients with MWS (n = 6) and age‑matched healthy controls (n = 10); ellipses indicate 95% confidence intervals. B, Permutation test (1000 permutations) to evaluate potential overfitting of the OPLS-DA model, showing the distributions of R^2^Y and Q^2^ across permuted models; the original model yielded R^2^Y = 0.862 and Q^2^ = 0.709, with a negative Q^2^ intercept (−1.84, p = 0.001) and a low R^2^ intercept (0.305, p = 0.004). C, Log_2_ fold changes of plasma neurotransmitters in patients with MWS versus healthy controls, highlighting significantly decreased serotonin, taurine and increased GABA, DOPA, and dopamine; bar colors denote significance levels (teal, p < 0.05; light red, p < 0.01; red, p < 0.0001).
